# Depression care integration in tuberculosis services: A feasibility assessment in Pakistan

**DOI:** 10.1111/hex.13985

**Published:** 2024-02-07

**Authors:** Saima Afaq, Aliya Ayub, Mehreen Riaz Faisal, Zara Nisar, Zala, Ateeq ur Rehman, Afzaal Ahmed, Olamide Todowede, Najma Siddiqi

**Affiliations:** ^1^ Department of Health Sciences University of York York UK; ^2^ Institute of Public Health & Social Sciences Khyber Medical University Peshawar Pakistan; ^3^ University of Nottingham Nottingham UK; ^4^ Hull York Medical School York UK

**Keywords:** depression, integrated care, LMIC, Pakistan, tuberculosis

## Abstract

**Background:**

The co‐occurrence of depression among tuberculosis (TB) patients is a critical issue, contributing to poor treatment outcomes, prolonged hospitalisations and increased healthcare expenses.

**Objective:**

The objective of this study was to assess the feasibility of delivering a co‐designed depression care pathway within TB services in Pakistan.

**Design:**

Mixed‐method study.

**Setting and Participants:**

Routine depression screening for TB patients was conducted at three TB facilities in Peshawar, Pakistan, encompassing primary, secondary and tertiary care settings. All patients aged 18 or above (male and female) attending the three TB facilities between November 2021 and February 2022 were included in the study using the consecutive sampling technique.

**Results:**

A total of 301 people with confirmed TB, within the past 4 weeks, visited the three TB care facilities; 191/301 patients were screened for depression. Approximately 35% of the 191 TB patients screened positive for depression, with varying severity levels. Qualitative findings highlighted the acceptability of integrated depression care, emphasising the importance of open communication and empathetic attitudes. Barriers to integration include stigma, logistical challenges, patient noncompliance and cost burdens. Facilitators included the empathetic attitude of healthcare providers and the availability of mental health services within the same facility.

**Conclusion:**

There is a high burden of depression in patients with TB, highlighting the pressing need for mental health support in this population. Acceptability of integrated care was evident, with factors such as co‐located mental health services, training healthcare providers and provider empathetic attitudes playing a crucial role. Further research is required to evaluate the effectiveness of the integrated TB‐depression screening systems towards improved health outcomes, implementation, scalability and impact on the broader healthcare system.

**Patient and Public Contribution:**

To create a more inclusive and comprehensive TB and depression care pathway, we gathered input from both service providers and service users (TB patients, their carers). Reflective meetings with community leaders, social activists and health professionals from various sectors were also conducted during pathway delivery to get their insights. Power, gender and age imbalances were addressed by encouraging participation of patients and carers across gender and age groups. This approach ensured that the perspectives of all stakeholders were considered in the development of the care pathway.

## 1 INTRODUCTION

The co‐occurrence of tuberculosis (TB) and depression presents a significant challenge in low‐ and middle‐income countries like Pakistan. The world's poorest populations are estimated to lose $12 billion of income each year due to TB,^1^ with the loss of productivity attributed, as estimated by the World Bank, to be 4%–7% of the gross domestic product in some countries.^2^ Pakistan is ranked fifth among high TB burden countries, recording over 510,000 new cases annually and an estimated 56,000 TB‐related deaths yearly.^3^, ^4^ Concurrently, depression rates are also high in Pakistan; 22%–34% of adults may have depression at some point, accounting for 3.13% of disability‐adjusted life years (DALYs).^5^, ^6^, ^7^

The association between depression and TB is well‐documented. Evidence from cross‐sectional studies and a systematic review, including studies from South Asia, show depression prevalence rates among TB patients ranging from 10% to 80.2%, with an average rate of approximately 50%.^8^, ^9^, ^10^ Studies from Pakistan also suggest that at least half of TB patients in Pakistan experience depression.^11^, ^12^ Furthermore, individuals with comorbid TB and depression often experience inferior treatment outcomes, prolonged hospitalisations and escalated healthcare expenses.^13^

Effective, integrated and scalable healthcare interventions are needed to address comorbidities.^14^ The World Health Organization (WHO) advocates for a person‐centred approach integrated across healthcare settings, emphasising coordination and alignment among various services.^15^ However, there is little evidence on how to achieve integrated care, especially in low‐ and middle‐income countries (LMICs). Recent Cochrane reviews highlight the uncertainty in the evidence regarding integration^16^ and recommend participatory approaches for developing interventions targeting specific comorbidity clusters such as TB and depression, using the Medical Research Council framework^17^ for the development and evaluation of complex interventions.^16^ The Academy of Medical Sciences emphasises the necessity for coordinated care, but it highlights a need for more research on how to coordinate or integrate care.^18^ Our study aims to bridge this significant knowledge gap by adhering to recommended methodologies, including a participatory approach to intervention development and delivery.

While Pakistan's TB control programme is well‐established, care for TB patients with comorbid depression lacks personalisation, leading to missed opportunities for screening, prevention and management. Synergies between TB and depression call for integrated models to effectively prevent, screen and treat both conditions. The WHO's End TB Strategy specifically advocates for integrating mental health services within TB care.^19^ Despite evidence‐based treatments available, Pakistan's national TB programmes face a significant treatment gap in mental health services. Existing TB‐related materials and tools offer no guidance on screening, preventing, managing or monitoring depression in TB patients. Additionally, evidence‐based personalised care packages for addressing depression within TB care are absent in the context of Pakistan.

The integration of depression care within TB services holds significant promise for addressing this treatment gap, reducing the disease burden and improving the health outcomes of people with TB.^8^ Given the established infrastructure of TB care in Pakistan, the integration of depression care presents a strategic avenue for enhancing healthcare delivery, thus warranting thorough investigation.

Khyber Pakhtunkhwa is in the northwestern province of Pakistan where approximately 55,000 new cases of TB (all types) are reported each year. During 2002–2017, more than 462,920 TB cases were reported in Khyber Pakhtunkhwa province,^20^ showing a higher TB prevalence than the rest of the country over the years.^21^ Additionally, a descriptive analysis of nationally collected data confirmed that notification of TB in Khyber Pakhtunkhwa is higher in females, contrary to global trends.^22^ The majority of female patients in higher notification districts were illiterate, unemployed, poor and living in households of low socioeconomic status when compared to women from low notification districts. These insights into the TB burden in Khyber Pakhtunkhwa underscore the urgency for integrated health strategies to help control the TB epidemic in the province more effectively. This study, therefore, aims to explore the feasibility of implementing a co‐designed depression care pathway within TB services in Khyber Pakhtunkhwa, Pakistan, focusing on coverage and acceptability among patients and healthcare staff.

This study is part of the Chronic Communicable Disease study nested within the IMPACT (Improving Mental And PhysiCal health Together in South Asia) programme,^23^ looking at the integration of depression care within TB services in Bangladesh, Pakistan and India.

## 2 MATERIALS AND METHODS

### 2.1 Study design and setting

This study was conducted to introduce and (pre and post) test the feasibility of routine screening for depression as part of usual care pathway for TB at three TB facilities in Peshawar, Khyber Pakhtunkhwa, Pakistan: (i) a district tuberculosis office (DTO)—a primary care setting, which is attended by an average of 608 patients per quarter; (ii) Government City Hospital—a secondary care setting, attended by an average of 262 patients per quarter; and (iii) Khyber Teaching Hospital (KTH)—a tertiary care setting, attended by an average of 217 patients every quarter. The study sites were identified based on the number of patients registered and treated. A readiness assessment of the facilities was performed using a checklist before the start of the study.

### 2.2 Sampling

All male and female patients aged 18 or above attending the three TB facilities were included in the study using the consecutive sampling technique. The recruitment timeframe was from 14 November 2021 to 14 February 2022. A patient information sheet and consent form, translated into the local language (Urdu and Pashto), were provided to the participants and written informed consent was obtained from those who wished to participate.

For qualitative interviews, we had two groups: service providers (the healthcare staff involved in delivering the care pathway) and service users (TB patients and their carers). The Directly Observed Treatment Short‐course (DOTS) programme facilitators, medical officers and the psychologist/psychiatrist from all three identified sites were invited for service providers' interviews. For the service users, convenience sampling was used where TB patients and their carers, who had been through the study process, were identified with the help of the DOTS facilitator and invited to participate in interviews via telephone at the end of the feasibility study.

### 2.3 Care pathway

The integrated TB and depression care pathway was co‐created through a series of ‘co‐design’ workshops with stakeholders across TB and mental health services, including TB patients, their carers and healthcare providers in Peshawar, Pakistan. To address the inherent power imbalances between healthcare providers and patients, as well as between genders and age groups, distinct workshops were conducted to cater to all genders and age groups within the relevant context. We encouraged participation of women and patients/carers who are usually less inclined to participate due to cultural factors (detailed co‐design workshop methodology to be published elsewhere).

**Figure 1:**
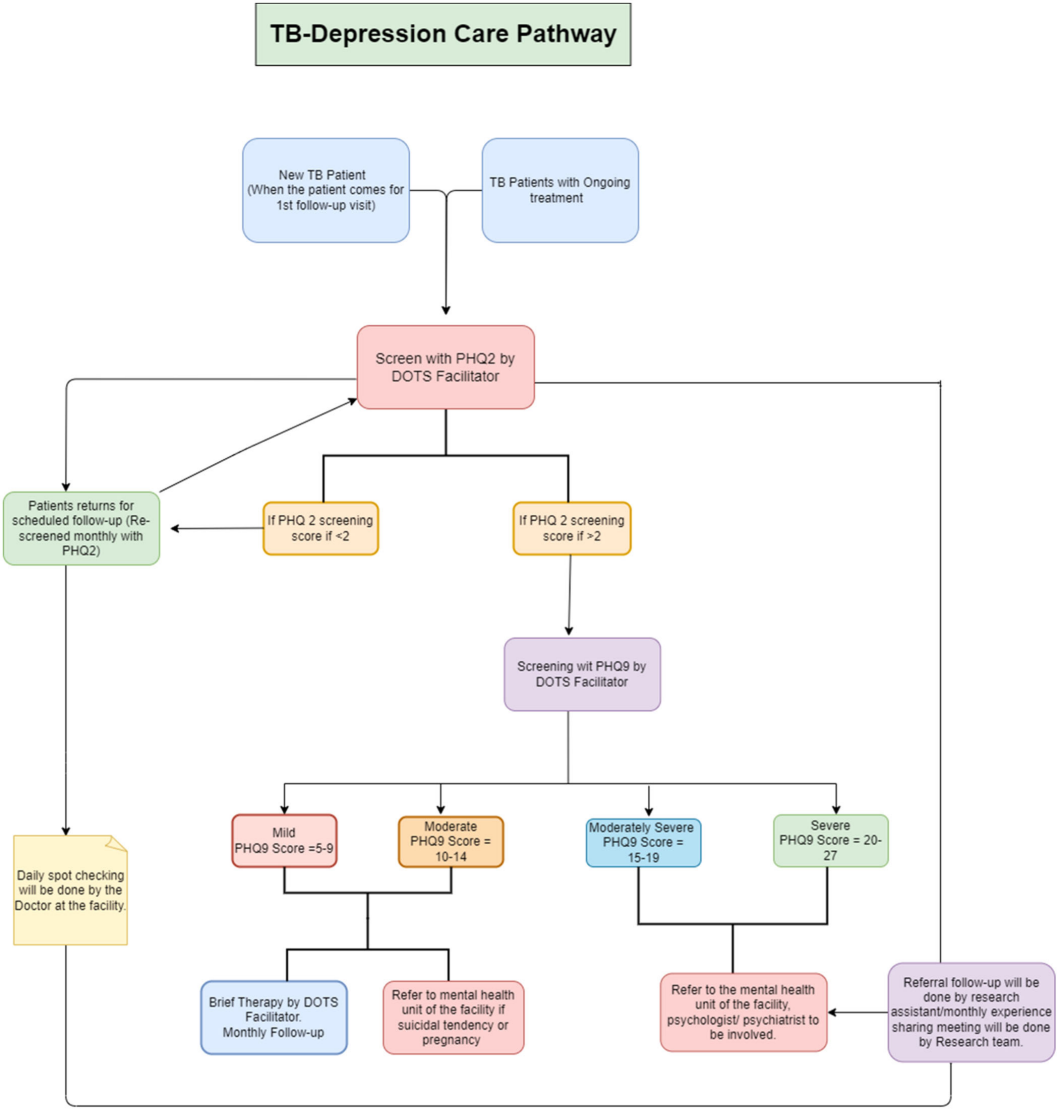
Tuberculosis (TB)—Depression care pathway for primary, secondary and tertiary TB facilities in Peshawar (Khyber Pakhtunkhwa), Pakistan.

Every adult patient (new and old) who visited the three study sites was screened for depression, regardless of previous depression status. All study sites were linked to a mental health facility for patient referral. As the first point of contact, the DOTS facilitator conducted the initial screening using the two‐item Patient Health Questionnaire‐2 (PHQ‐2) by Kroenke et al.^24^ Patients with a score of 2 or less entered the facility's usual care pathway, whereas those with a score of more than 2 were further screened using the nine‐item Patient Health Questionnaire‐9 (PHQ‐9) by Kroenke et al.^25^ Patients were further managed based on their PHQ‐9 screening scores: no depression (0–4), mild (5–9), moderate (10–14), moderately severe (15–19) and severe (20–27) (Figure 1).

Patients with a score of 0–4 (no depression) followed the facility's usual care pathway. Patients with a score of 5‐9 (mild) and 10–14 (moderate) were counselled and reassured by the DOTS facilitator. The DOTS facilitators were trained following the WHO's Mental Health Gap Action Programme (WHO's mhGAP) module, which includes content for nonmental health specialists to provide mental health support to these patients. For the convenience of the DOTS facilitators, a brief counselling outline was provided to them, including reassurance to the patient about the disease's cure, information on the side effects of the medications and contact information for providing any help when needed.

Patients with a score indicating moderately severe (10–14) and severe depression (15–19), and those who required immediate attention, such as suicidal ideation or those pregnant, were referred to mental health specialist facilities. The nearest mental health specialist facility was linked during the pathway co‐design for the primary care setting. In contrast, for secondary and tertiary care settings, referrals were made to the mental health department of the same facility. The medical officer or facility manager signed the referral slip at each study site.

### 2.4 Data collection

#### 2.4.1 Quantitative data

Data collection tools provided to the DOTS facilitators included (1) consent forms, (2) screening proforma, (3) PHQ‐2 Slip, (4) PHQ‐9 Slip and (5) referral slip. In addition, data on sociodemographic factors was captured by the site research assistants. Data was entered into an online survey tool (Qualtrics) by the research assistant at each site. Predata collection training was provided to the TB care facility staff (DOTS facilitator, medical officer and the manager), which included: (1) screening, recognition of depression and treatment of depression (using PHQ‐2 and PHQ‐9) according to the mhGAP curriculum, (2) understanding the codesigned TB and depression integrated care pathways including referral for specialist advice and (3) study data collection procedures and materials.

#### 2.4.2 Qualitative data

A semistructured topic guide was used during the focus group discussions (FGDs). It included questions on the acceptability of integrating depression screening and referral in the TB care pathway, barriers and facilitators and any suggestions the participants may have after having experienced the process. Prompts were used appropriately to explore further any issues the participants expressed.

Trained research assistants conducted FGDs either at the study sites or at Khyber Medical University Peshawar, as per the feasibility of the participants. All FGDs were audio recorded with the consent of participants and transcribed by the research assistants. The transcriptions were anonymised. The sampling frame is provided in Table 1.

**Table 1:** Sampling frame (demographics) of focus group participants.

Session	Number of participants (*n*)	Gender
Focus group 1 (service users)	Total participants = 5	Male = 2 Female = 3
	Patients = 3	
	Carers = 2	
Focus group 2 (service users)	Total participants = 3	Male = 1 Female = 2
	Patients = 1	
	Carers = 2	
Focus group 3 (service users)	Total participants = 5	Male = 2 Female = 3
	Patients = 3	
	Carers = 2	
Focus group 4 (service providers	Total participants = 6	Male = 5 Female = 1
	Psychiatrist = 2	
	Pulmonologist = 2	
	Pulmonologist = 2	
Focus group 5 (service providers	Total participants = 6	Male = 5 Female = 1
	Psychiatrist = 2	
	Pulmonologist = 2	
	DOTS facilitator = 2	

Abbreviation: DOTS, Directly Observed Treatment Short‐course.

### 2.5 Data analysis

Quantitative data was analysed using the Statistical Package for the Social Sciences (SPSS version 23 software). A descriptive analysis was conducted. Mean and standard deviations were reported for continuous data (PHQ‐2 score and PHQ‐9 score) and frequencies and percentages for categorical data.

The thematic analysis outlined by Braun and Clarke^26^ was followed to analyse the qualitative data. This approach entails searching and analysing patterns in the data set^27^ through a six‐step process: data familiarisation, generating initial codes, searching for themes, reviewing themes, defining and naming themes and producing the report.^26^ Two coders (Z. N. and M. R. F.) independently coded the data using inductive and deductive approaches. Regular meetings were held to discuss the codes and themes generated.

## 3 RESULTS

### 3.1 Sociodemographic profile of people with TB

Between November 2021 and February 2022, a total of 301 people with confirmed TB (within the past 4 weeks) visited the three TB care facilities: 168 in DTO, 72 in Government City Hospital and 61 in KTH, based on the patients' flow. A total of 191/301 (63.45%) patients were screened for depression using PHQ‐2. The remaining 110 patients refrained from physically attending the study sites, opting instead for medication collection facilitated primarily by their relatives. Consequently, these patients were not subjected to the screening process. Out of the 191, the primary care facility screened 62 (32.3%), the secondary care facility screened 65 (33.9%) and the tertiary care facility screened 64 (33.3%) people, respectively. An almost equal proportion of males and females were screened (52.9% and 47.1%, respectively). Over half of the participants were married (56.8%). Most participants were nonsmokers (94.3%), while approximately 2% took other addictive substances. Around two‐thirds (70.7%) of participants had pulmonary TB in contrast to the remaining third (29.3%) who suffered from extrapulmonary TB (Table 2).

**Table 2:** Characteristics of the participants visiting the TB facilities and screened for depression (*n* = 301).

TB patients characteristics	*N* = 191 (%)
Age (years), (mean (SD)	33.01 (15.23)
Gender	
Male	(101) 52.9%
Female	(90) 47.1%
Level of education	
None	101 (52.6%)
Informal education	2 (1.6%)
Primary	21 (10.9%)
Secondary	35 (18.2%)
Postsecondary (tertiary)	32 (16.7%)
Employment status	
Employed	85 (44.5%)
Unemployed	25 (13.1%)
Student	20 (10.5%)
Unable to work	5 (2.6%)
Housewife	56 (29.3%)
Marital status	
Married	108 (56.8%)
Single	81 (42.2%)
Widowed	2 (1%)
Smoking status	
Smokers	10 (5.2%)
Nonsmoker	181 (94.8%)
Level of depression	
People with TB screened using PHQ‐2	191 (63.4%)
People with TB with PHQ‐2 score >2	55 (28.8%)
People screened with PHQ‐9	55 (100%)

Abbreviations: PHQ, Patient Health Questionnaire; TB, tuberculosis.

### 3.2 Sociodemographic profile of the focus group participants

Five FGDs were conducted with service providers and service users. These included two sessions with six healthcare professionals and three with patients and carers. The focus groups were conducted over 2 months in March and April 2022, with each session lasting for 50 min on average. The characteristics of the focus group participants are presented in Table 1.

### 3.3 Depression rates among people with TB

**Figure 2:**
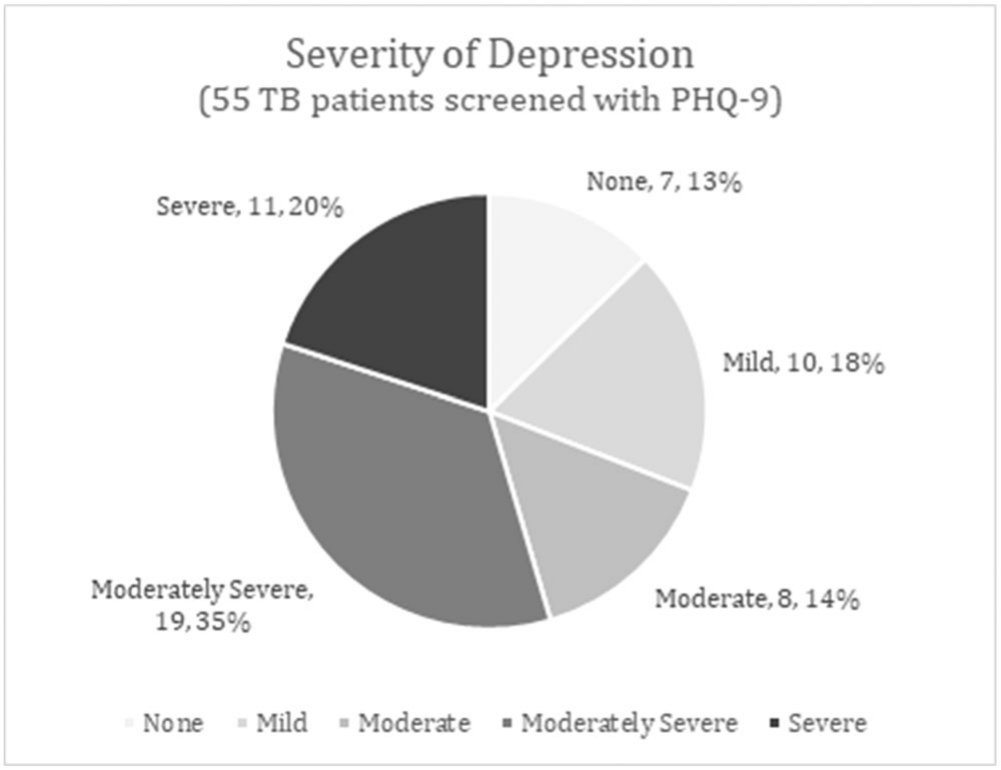
Severity of depression (55 tuberculosis patients screened with Patient Health Questionnaire‐9).

Amongst the 191 participants screened for depression using PHQ‐2, 28.8% (55) scored >2 and were further screened using PHQ‐9. Screening using PHQ‐9 confirmed that 13% did not have depression, while the proportion of participants with mild, moderate, moderately severe and severe depression was 18%, 14%, 35% and 20%, respectively (Table 2 and Figure 2). All participants with moderately severe and severe depression were referred to specialised mental health facilities.

### 3.4 Barriers and facilitators to integration of depression care in TB care

Thematic analysis of the data was undertaken, and subthemes were categorised under the four overarching themes of (1) acceptability of integration of depression care in the TB care pathway, (2) barriers, (3) facilitators for integration of care and (4) suggestions for improvement of the programme. Illustrative quotes are presented in Table 3.

**Table 3:** Illustrative quotes of participants from focus groups.

Participant ID	Quote
P1/002—Service user	Yes, their words are very encouraging. They don't let us feel like any stigma is attached to the disease. I felt embarrassed by having TB, but the person who counselled me said it was not my fault and it was a treatable disease. Hence, we understand that we are improving and this disease is curable.
P1/003—Service provider (DOTS facilitator)	Yes, in the beginning, everything seemed difficult. Still, with proper counselling and inculcating a sense in patients, all these benefit them and can prove a milestone in achieving the target.
P3/004 and P2/004—Service providers (DOTS facilitators)	P3: Initially, the patient was unaware of this screening, so we faced hurdles because instead of the patient, the patient's attendant would collect the medicine on their behalf. But then we applied a different strategy for the patient to come to OPD for their own medicine. We contacted them by phone and called them to the facility. So, after this, the screening process got smooth. P2: Exactly; before this screening process, patient attendants would come with the original CNIC of the patient to collect medicine every month and patients visit only after three or four months for repeat investigations, so it was hard to screen them, but then we asked attendants to bring a patient to OPD so you can get medicine otherwise we are not allowed to give you medicine without the patient.
P1/003—Service provider (DOTS facilitator)	Well, the main issue we endured was that both wards were on different floors. Patients found it difficult to visit upstairs.
P2/004—Service provider (DOTS facilitator)	One thing I heard from patients is that they cannot afford antidepressants because of their low socioeconomic background.
P1/001—Service user	Q: Did you take the medicines your psychiatrist prescribed? P1: No, I didn't use those medicines. Q: Why? Can you please tell me? P1: These medicines are not right. How can the doctor tell without taking my history? So, after I told Dr. A, he referred me to another psychiatrist; from his medicines, there was a lot of hair loss, and I visited again.
P4/004—Service provider (psychiatrist)	Yes, definitely, it would be a great initiative because, in our society, mental health is ignored. One in three persons is depressed, but mental health is not common and people are unaware of mental disorders. They associate these disorders with other morbidities or ignore them. Adding this step to screening processes would be a great relief for improving society's productivity.
P2/004—Service provider (DOTS facilitator)	In our hospital system, there are many facilities for patients under one roof. Examination of TB patients and screening was easy. Patients regularly come for their medicines. We got to the psychiatry unit near our OPD. There was no problem with the referral system.
P1/001—Service user	The psychiatrist needs improvement. He was not focusing as we entered the room and sat, so he asked only two questions. As I told you, 50% of doctors didn't give their proper time to patients. While in 50%, only 2% of doctors are like Dr. A, and they listen to their patients properly.
P1/003—Service provider (DOTS facilitator)	All I can suggest is to bring the whole activity to one floor so that it becomes easy to fulfil every step.
P4/004—Service provider (psychiatrist)	This process should start from the basic health unit (primary health care). It would be a great initiative to screen depressed patients at every level and help increase mental health awareness and education.

Abbreviations: DOTS, Directly Observed Treatment Short‐course; OPD, out‐patients department.

#### 3.4.1 Theme 1: Acceptability of integration of depression care in TB care pathway

Overall, the service users and providers found the depression screening and referral service acceptable. The service users commented about being pleasantly surprised to find someone willing to discuss their problems. Given the stigma attached to health conditions such as TB and mental health, the responses from the service users highlighted instances in which healthcare professionals' effective communication skills with an empathetic attitude can significantly facilitate the engagement of TB patients for depression screening and care services (P1/002, service user).

However, service providers spoke about instances when patients seemed reluctant to be involved. They had to educate the patients about depression and why screening is necessary before they were able to proceed (P1/003, service provider [DOTS Facilitator]).

#### 3.4.2 Theme 2: Barriers to integration of depression care in TB care pathway

For the barriers to integration, the service providers referred to barriers related to screening and referral for further evaluation. Although the stigma around mental health conditions was spoken about as a challenge, service providers felt that after educating the patient, they could overcome this barrier.

However, patients not attending the follow‐up visits were highlighted as an essential barrier to depression screening. It is a common practice for patients to send someone, such as a family member or a friend, on their behalf for the TB medication, which is to be collected every month. Nevertheless, the service providers spoke about overcoming this barrier, to a large extent, by contacting the patients through the telephone or their attendants, asking them to visit themselves for their medication (P3/004 and P2/004, service providers [DOTS facilitators]).

Other barriers to screening that were reported included logistical challenges such as lack of capacity due to work burden/shortage of staff, screening set up on a separate floor to where the patients attended the out‐patients department, and nonavailability of lift access (P1/003, service provider [DOTS Facilitator, Naseer Ullah Babar, hospital]).

Service users and providers spoke about challenges related to referrals for further evaluation to a mental health facility and patient compliance with the referrals. These included the distance to be travelled to be seen by a mental health professional and the cost of anti‐depressant medications (P2/004, service provider [DOTS facilitator]).

Additionally, service users felt a sense of mistrust when the mental health professionals failed to give them enough time or did not listen attentively to their concerns, which could have further added to noncompliance with referrals (P1/001, service user).

#### 3.4.3 Theme 3: Facilitators to integrate depression care in the TB care pathway

The service users spoke about the health professionals' empathetic attitude and good communication skills as facilitators for the programme (P1/005, service user).

The service providers felt that good coordination between the TB care and the psychiatry unit facilitated the smooth running of the programme. Furthermore, health professionals' positive attitude, including going the extra mile to contact patients by telephone to encourage them to visit, and willingness to incorporate depression screening as part of TB services, were also found to be facilitators for future implementation of the programme (P4/004, service provider [psychiatrist]).

In one of the TB care sites, psychiatric services were offered under the same roof, thus precluding the need for patients to travel long distances to consult a mental health professional. This greatly facilitated the patient referral process (P2/004, service provider [DOTS facilitator]).

#### 3.4.4 Theme 4: Suggestions for improvement

The service users highlighted the importance of effective communication skills among healthcare providers, emphasising the importance of active listening and an encouraging attitude (P1/001, service user).

The service providers shared suggestions for the highlighted barriers, including providing TB care and mental health services under one roof and subsidising medication costs to reduce cost barriers around antidepressant medication (P1/003 and P2/004, service provider [DOTS Facilitators]). Furthermore, the service providers also spoke about providing mental health training for nonmental health specialists and the scale‐up of depression screening to include other services and primary care to raise awareness regarding the importance of mental health and related problems (P4/004, service provider [psychiatrist]).

## 4 DISCUSSION

Our findings show that around 35% of the people with TB had moderately severe depression and another 20% had severe depression when screened using PHQ‐9. Overall, there was an indication of the acceptability and usefulness of the service by both service users and the service providers. However, some of the barriers encountered were related to logistical challenges such as lack of space and privacy and increased workload for the staff. Other reported barriers included patient noncompliance, stigma associated with mental health conditions, mistrust, cost burden for the patient regarding the cost of medicines and time and money spent travelling to a mental health facility. Facilitators included the empathetic attitude of health professionals and the availability of mental health services under the same roof for one of the study sites. Furthermore, there were suggestions to extend the service to other health facilities, including primary care, provision of mental health training to nonmental health specialists and reducing cost barriers for service users to obtain medication.

Our findings indicate that approximately half of the screened individuals with TB were depressed. These results align with previous studies conducted in Pakistan.^11^, ^28^ The bidirectional link between these illnesses could explain the significant depression rate among TB patients. TB can cause psychological distress and depression due to the social stigma associated with the disease, fear of transmission to others and the impact of therapy on everyday living.^29^ This study emphasises the importance of addressing TB‐related stigma and advocates for an integrated approach that combines clinical services with a strong social support system that involves family and community to improve holistic, person‐centred care as recommended by the previous research.^30^ Furthermore, physical signs of tuberculosis, such as cough, fatigue and weight loss, may contribute to emotional distress.^31^, ^32^ Depression, conversely, can decrease the immunological response, increasing vulnerability to TB infection and delaying recovery.^29^

The implications of the high occurrence of depression among TB patients are substantial. Depressed TB patients are more likely to have poor adherence to TB treatment, which leads to treatment failure, relapse or development of drug resistance.^29^, ^33^ This compromises individual patient outcomes and contributes to the community's total TB burden. Furthermore, depression can adversely affect patients' quality of life and capacity to participate in everyday activities and jobs, leading to additional socioeconomic implications. However, a systematic review^34^ shows that psychoemotional support interventions can positively impact TB treatment outcomes.

Our research also explored the barriers and facilitators to integrating depression care within TB services. Both service users and providers considered the integrated approach acceptable, emphasising the importance of open communication, empathy and nonstigmatising attitudes in developing trust and encouraging patients to engage with mental health support. This aligns with current literature, emphasising patient‐centred treatment's importance in increasing acceptance.^8^, ^35^ The positive reaction to our integrated approach strengthens the possibility of addressing TB patients' mental health issues within their present care framework. Furthermore, training nonmental health specialists, such as DOTS facilitators, in providing mental health support can improve mental health service accessibility within TB care facilities.^8^, ^36^

The stigma associated with mental health disorders, logistical challenges and economic constraints were identified as potential barriers to integrating depression and TB care. While some of the barriers identified are similar to those identified in previous research from LMICs, where healthcare facilities, specifically mental health facilities, frequently struggle with issues related to being under‐resourced and receiving inadequate prioritisation,^8^, ^37^ this study provides insights into local differences and specific challenges faced in the integration of depression care within TB services in Pakistan. Patients may avoid seeking help due to a lack of access to mental health care and the accompanying costs, resulting in untreated depression. In our study, referral barriers and noncompliance were indicated as significant issues. The practice of sending representatives to collect medication may explain patients' reluctance to self‐attend TB care facilities for screening. This behaviour highlights the importance of exploring ways to increase direct patient involvement and compliance, such as personalised phone calls and reminder systems, as well as active systems to measure referral uptake.^38^, ^39^ In addition, activities focused on increasing awareness, education and community involvement are critical to promoting the acceptability of interventions in these patients.^40^, ^41^

Our findings agree with the Global Mental Health movement,^42^ the aims of which directly align with integrating mental health services into existing TB care programmes, eventually providing service delivery within nonspecialised psychiatric settings. In low‐resource settings, like Pakistan, with few mental health specialists, this may be achieved through ‘task shifting’. Task‐shifting, which enables nonspecialist healthcare professionals to provide mental health support, has improved access to care in resource‐constrained settings.^43^ Likewise, collaborative care strategies that promote collaboration between mental health and TB care professionals have been reported to effectively utilise the limited resources in LMIC settings.^44^, ^45^ Such methods can provide complete care that addresses physical and mental health together.

Building TB staff capacity and training is essential to achieving integrated depression and TB care. Solutions for capacity building of nonmental health specialists are now increasingly available. Mental health service provision is explicitly included in the TB eradication strategy pillars by the WHO's End TB mandate.^46^ We used the WHO mhGAP^47^ with an evidence‐based mental health intervention package for depression for training the DOTS facilitators in our study. While not explicitly designed for TB, these interventions could be adapted to the specific needs of patients with TB and used as a first line for training nonmental health specialists and integrating mental health services in TB facilities. Other global organisations, such as The Union^48^ have developed similar guidelines. These organisations can play a significant role in educating relevant stakeholders through disseminating educational materials.

An important first step for the TB programme would be to scale up routine screening of common mental disorders, including depression. Although similar screening methodologies have been reported in different settings,^49^, ^50^, ^51^ the implementation in the specific context of TB care in Khyber Pakhtunkhwa province, Pakistan, is novel and has not been previously implemented. We tailored previously established approaches to address the unique challenges faced by TB patients in our setting. Furthermore, our study identified the specific roles of healthcare providers involved in delivering integrated care within TB care facilities, which has not been previously explored in the low‐resource setting of Pakistan with fragmented health systems. With only two (8%) of 26 countries reporting the availability of routine mental health screening in their TB programmes,^46^ introducing a simple and short screening tool for depression, such as the PHQ‐9, would be a significant step towards characterising depression among people with TB and providing TB programmes with the data needed to leverage funding for mental health services. Furthermore, implementing a stepped‐care approach for depression management^52^ could benefit TB care facilities where low‐intensity interventions, such as counselling and psychoeducation, are provided as a first step, followed by more specialised interventions for patients with severe symptoms. Stepped‐care models^52^ are effective in improving mental health outcomes while optimising resource utilisation.

The implementation of the collaborative co‐design algorithm for addressing both TB and depression has proven effective in the identification and management of depression among TB patients. Our approach involved comprehensive training programmes for TB health professionals, equipping them with the necessary skills to proficiently screen and identify depression cases using validated measures, as well as to manage or refer those cases to specialised care as required. Our iterative implementation feasibility study aimed not only to bridge the gap in mental health care for TB patients but also to ensure that these efforts were supported and sustained by continuously refining the pathway during its implementation. We conducted regular reflective meetings, creating a space to address barriers and challenges, seek collaborative solutions and improve the overall efficiency of the integrated care pathway. The active involvement and endorsement of health professionals as evident in the qualitative interviews further demonstrate the potential for sustained success and scalability of this intervention. This integrated approach, supported by collaborative efforts and continuous refinement, stands as a promising model for improving contextualised person‐centred care for addressing mental health concerns among TB patients, setting the foundation for future healthcare interventions.

## 5 STRENGTHS AND LIMITATIONS

We included complete patient screening (using both PHQ‐2 and PHQ‐9) in primary, secondary and tertiary TB care settings. Having locally translated versions of the PHQ‐2 and PHQ‐9 questionnaires allowed accessibility for various groups. Expert training sessions facilitated the implementation of the care pathway. Regular reflective sessions were critical in identifying and resolving difficulties in the integrated care pathway, increasing overall acceptance by service providers and users.

The study has some limitations. There may be limited generalisability of the findings as the study was conducted in three sites only and was of relatively short duration. Furthermore, as the study included a pre–poststudy design, we involved all drug‐sensitive TB patients attending the TB facilities during the study period and did not undertake any formal sample size calculations. A comprehensive monitoring and evaluation strategy could not be implemented due to time and financial restrictions. Therefore, future studies should assess the effectiveness of the TB and depression care pathway through fully powered stepped‐wedge randomised trials, ensuring sequential access to integrated care while offering a fair evaluation of its effectiveness across participating clusters.

## 6 CONCLUSION

The significant prevalence of depression among TB patients underscores the urgent requirement for mental health assistance within this group. The integration of care has demonstrated its acceptability, with factors such as the proximity of mental health services, the training of healthcare providers and their empathetic attitudes playing vital roles. Further investigation is necessary to assess the effectiveness of this integrated approach in enhancing health outcomes, its implementation, scalability and its impact on the broader healthcare system.

## AUTHOR CONTRIBUTIONS

**Saima Afaq**: Conceptualisation; methodology; validation; writing—review and editing; writing—original draft; supervision; data curation; software; project administration; resources; funding acquisition; formal analysis; visualisation. **Aliya Ayub**: Data curation; visualisation; writing—review and editing; investigation. **Mehreen Riaz Faisal**: Formal analysis; writing—review and editing; supervision; validation. **Zara Nisar**: Data curation; formal analysis; project administration; writing—review and editing; supervision; validation. **Zala**: Formal analysis; visualisation; writing—review and editing. **Ateeq ur Rehman**: Writing—review and editing; investigation. **Afzaal Ahmed**: Writing—review and editing; investigation. **Olamide Todowede**: Conceptualisation; methodology; validation; writing—review and editing. **Najma Siddiqi**: Conceptualisation; methodology; writing—review and editing; supervision.

## CONFLICT OF INTEREST STATEMENT

The authors declare no conflict of interest.

## ETHICS STATEMENT

The study was approved by the University of York's Health Sciences Research Governance Committee (HSRGC/2020/418/D). Ethical approval was also obtained from Khyber Medical University Pakistan (Dir/KMU‐EB/IPHSS/MM‐001). Written informed consent was received from all the participants.

## Data Availability

The data will not be uploaded to a public repository due to the sensitivity of the information. The data, however, is available upon request to the corresponding author.
